# Computational protein design with backbone plasticity

**DOI:** 10.1042/BST20160155

**Published:** 2016-10-19

**Authors:** James T. MacDonald, Paul S. Freemont

**Affiliations:** 1 Centre for Synthetic Biology and Innovation, South Kensington Campus, London SW7 2AZ, U.K.; 2 Section of Structural Biology, Department of Medicine, Imperial College London, London SW7 2AZ, U.K.

**Keywords:** computational protein design, conformational sampling, flexible backbone design

## Abstract

The computational algorithms used in the design of artificial proteins have become increasingly sophisticated in recent years, producing a series of remarkable successes. The most dramatic of these is the *de novo* design of artificial enzymes. The majority of these designs have reused naturally occurring protein structures as ‘scaffolds’ onto which novel functionality can be grafted without having to redesign the backbone structure. The incorporation of backbone flexibility into protein design is a much more computationally challenging problem due to the greatly increased search space, but promises to remove the limitations of reusing natural protein scaffolds. In this review, we outline the principles of computational protein design methods and discuss recent efforts to consider backbone plasticity in the design process.

## Introduction

A variety of different strategies have been developed to engineer novel globular proteins. These range from directed evolution, simple residue patterning methods, to atomic-level computational protein design. There has been less progress in the design of membrane proteins due to the difficulty in experimental characterisation [1], so this review concentrates mainly on the design of globular domains. Directed evolution methods are well established and have produced notable successes [2]. These methods generally require a starting protein sequence with some initial activity from which to generate and select variants. Mutations that increase the desired activity may be very rare, requiring high-throughput screening. The rational design of proteins using residue patterning has been particularly successful in the design of *de novo* helical bundle proteins [3], self-assembling coiled-coil peptides [4], and repeat proteins [5]. These proteins have been functionalised by intuitive manual design to introduce chemical activity [6,7].

**Figure 1.**
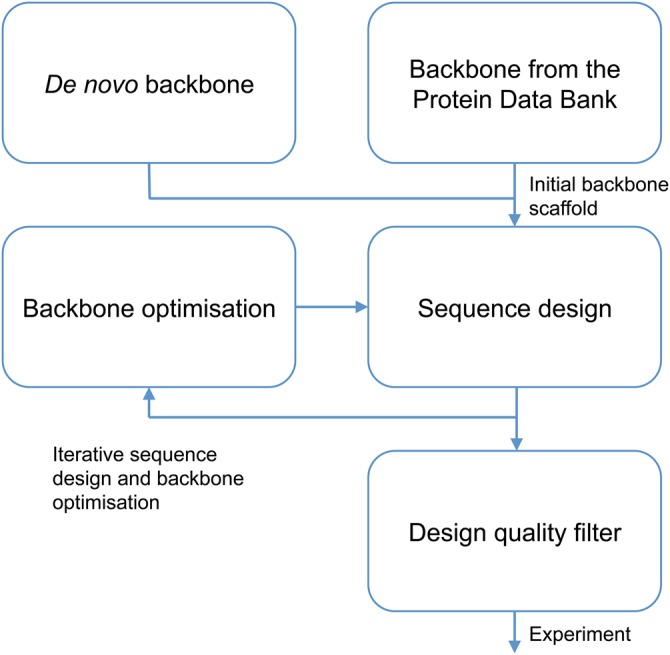
A typical computational protein design workflow. Initial backbone structures can be either generated *de novo* or taken from solved protein structures. Sequences that stabilise the designed backbone structure are then computationally designed, and the backbone may be permitted to move as part of an iterative design cycle. Finally, promising designs are selected for experimental characterisation.

In contrast with the previously described methods, computational protein design algorithms construct detailed full-atom models. The ability to place chemical moieties with atomic-level precision enables applications not possible with other protein engineering methods. Initial computational protein design work focussed on finding optimal sequences for fixed-backbone scaffolds taken from natural proteins [8–10]. These fixed-backbone computational design algorithms have been extended to introduce novel functionality, such as binding sites [11], libraries of fluorescent proteins [12], and *de novo* designed enzymes that catalyse reactions not found in nature [13,14]. In parallel to these developments, entirely *de novo* proteins, consisting of mainly canonical secondary structure and minimal loops, have been created by assembling backbone fragments from known protein structures, followed by iterated sequence design using the fixed-backbone approximation and energy minimisation [15–17]. However, in general, the rules, governing the designability of a given arbitrary backbone conformation, are not well understood. An outline of a typical computational protein design process is shown in Figure 1.

Given the rough landscape of full-atom potential energy functions, sequence design on a fixed-backbone artificially restricts the possible amino acid residues capable of being accommodated at a given position. Even small changes in backbone conformation may permit residues that were previously sterically hindered, and therefore improve the diversity in designed sequences [18]. The incorporation of larger scale backbone plasticity in computational designs will also allow further optimisation of engineered proteins [19], and the greater freedom will allow the implementation of more complex functionalities. For these reasons, flexible backbone protein design is an increasingly important area of research.

## Computational protein design with the fixed-backbone approximation: the inverse folding problem

Computational protein design can be considered to be composed of two linked problems. The first problem is selecting or generating a plausible and designable backbone conformation. The second problem is finding sequences capable of specifically stabilising that backbone structure, also known as the inverse folding problem. The latter of these two problems will be discussed in this section.

Most modern methods use full-atom models of protein structure and molecular mechanics potential energy functions, consisting of a sum of covalent and non-covalent terms. These potential energy functions are often derived from force-fields developed for molecular dynamics simulations and may be supplemented with additional statistically derived terms [20]. Given a fixed-backbone, only side-chain identities and conformations are allowed to vary. Discrete libraries of side-chain conformations, known as rotamers [21], are commonly used to simplify the sampling and computation of potential energies. While the potential energy functions used in protein design are far from perfect, it has been observed that structures close to the native state almost always have the lowest potential energies, and it appears that conformational sampling is the bigger problem [22,23].

Stable proteins have a large energy gap between their native structure and all other possible structures. To rigorously determine whether a particular sequence specifically stabilises a given conformational state, it is necessary to evaluate the potential energy of that sequence over all possible (backbone and side-chain) conformations to calculate the partition function (i.e. the normalising constant required to ensure a probability distribution sums to 1). It is, then, possible to calculate the probability of any particular conformational state being occupied using the Boltzmann distribution (eqn 1, where *P_i_* is the probability of the system being in state *i*, *E_i_* is the energy of state *i*, *k* is the Boltzmann constant, *T* is the temperature, and the denominator is the partition function, a sum over all states). Optimising this probability by searching sequence space would then solve the protein design problem for a given backbone conformation. Unfortunately, this is computationally intractable, so, in practice, a variety of approximations have been used. Most common methods approach this problem by optimising a potential energy function by trialling different side-chain identities and rotamers without explicitly considering alternative backbone conformational states. It has been proposed that it is less important to consider alternative conformations in three dimensions as most low-energy decoys will have dissimilar structures, making mutations that stabilise the native state unlikely to stabilise the other conformations [24]. Side-chain identity and rotamer search may be conducted by deterministic methods, such as dead-end elimination [25,26], or by stochastic methods [27–29].

1 $$P_{i}=\frac{e^{(-E_{i})/kT}}{\sum_{j=1}^{N}e^{(-E_{j})/kT}}$$

Previously, it was found that proteins designed using hydrophobic patterning methods, did not seem to fold into well-defined native states and appeared to be more similar to molten globules. For this reason, early computational work concentrated on improving the specific packing in the hydrophobic cores of proteins [8]. This was followed by the ground-breaking automated sequence redesign of an entire small protein [9]. A larger scale test showed that this approach could successfully produce well-folded proteins by redesigning a range of different proteins [10]; however, it was notable that the redesigned proteins composed primarily of β-sheets appeared to be aggregated or unfolded.

The fixed-backbone assumption has proved to be sufficient to successfully create proteins with novel functionality without considering backbone flexibility. If a constellation of side-chain chemical groups can be defined that are predicted to carry out a given function (e.g. a transition state model), it is then possible to search existing protein structures for backbone positions capable of hosting this geometric arrangement, while taking into account side-chain degrees of freedom and steric clashes. Various algorithms have been developed to accomplish this [30,31]. The RosettaMatch algorithm uses an ‘outside-in’ approach by constructing the transition state model at the ends of each catalytic side-chain rotamer at all possible positions in the scaffold and recording the six-dimensional position of the transition state model in a hash table. If all catalytic geometric constraints can be satisfied simultaneously with a given selection of residue positions, this would result in the transition state model being reconstructed in the same position from all catalytic side-chain residues. Hits can be rapidly determined by scanning the hash table [31]. This algorithm enables the search of a large database of potential scaffolds, and its utility was dramatically demonstrated by the successful design of *de novo* enzymes using theoretic transition state models [13,14].

## Backbone sampling methods in protein design

Despite the achievements of fixed-backbone design, it is clear that this approximation is not sufficient to accurately sample sequence space and, more importantly, greatly limits the opportunities to optimise functional interactions. Backbone motion is also known to be functionally important in many natural proteins in molecular recognition [32,33] and enzymes [34].

The active site search algorithms described in the previous section are only able to search putative scaffolds for three to four catalytic residue geometries which are probably not enough to recapitulate extraordinary catalytic activities of natural enzymes [35]. The ability to redesign backbone structures around the catalytic site is likely to offer opportunities to optimise enzymes in ways that are not available to fixed-backbone approaches. In a previous paper, Foldit players were able to redesign a 24-residue backbone section and increase the activity of an artificially designed Diels–Alderase enzyme >18-fold [19].

The consideration of backbone plasticity in protein design requires the sampling of both backbone conformational space and side-chain identities/rotamers, and this enormously expands the search space. Additionally, unlike side-chains, backbone conformations are not amenable to discretisation. For these reasons, initial work on flexible backbone design was based on parameterised coiled-coil backbones [36], rigid body movements of secondary structural elements [37], and the introduction of small random backbone dihedral angle perturbations during the design process [38].

In parallel to the advances in computational protein design, a number of groups working in the related field of protein structure prediction found that short backbone fragments taken from previously solved protein structures could be used to explore backbone conformational space in an efficient way [39]. The backbone fragments are defined in terms of internal dihedral co-ordinates then as part of a Monte Carlo search procedure, random sections of the backbone are replaced with dihedral angles from the fragment in process called ‘fragment insertion.’

In an extraordinary achievement, this fragment insertion process was used to assemble an entirely *de novo* backbone fold not observed in nature with a computationally design sequence. A high-resolution crystal structure confirmed that the protein did indeed fold into the designed structure with atom-level precision [15]. This approach was subsequently generalised and extended to other folds using emergent rules [16,17]. These *de novo* folds consisted of idealised secondary structural elements linked with loops of minimal length. The use of existing backbone fragments in this way ensures that the local structural features of the designed protein replicate those observed in real proteins and increases the chance that the new backbone structures are designable. Recently, these computational design methods have been successfully applied to the design of more complex artificial coiled-coil proteins [40–43].

Fragment insertion is a non-local move as replacing dihedral angles in a particular backbone segment results in a move that propagates down the entire polypeptide chain. This is an inherently highly disruptive move, resulting in low acceptance rates in Monte Carlo simulations. However, fragment insertion can be turned into a local move by combining it with methods that can close chain breaks. A fragment is inserted midway along the chain, and a break is introduced at the N- or C-terminal end of the insertion so that the rest of the polypeptide chain is not moved. Adjustments then need to be made to the dihedral angles in the fragment such that the chain recloses. There are various algorithms that solve this loop closure problem, many of which are related to methods used to control robotic arms. These include cyclic co-ordinate descent (CCD), where each backbone dihedral angle is optimised in turn until the correct geometry at the break is restored [44], kinematic closure, where all dihedral angles in the loop may be freely varied except six dihedrals which are solved for loop closure using polynomial resultants [45], and stochastic closure methods [46].

Fragment-based approaches have been used to computationally design loop structures on natural protein scaffolds. By selecting backbone fragments from the PDB with endpoints that superimpose with the anchor residues in the scaffold, Hu et al. were able to graft 10-residue loops on to the protein tenascin. The inserted loops ranged from 0.9 to 1.6 Å backbone RMSD from the wild-type loop. The loop endpoints were close enough to the anchor residues that the loops could be closed by gradient minimisation. Two loops were solved using X-ray crystallography, and one was found to match the designed loop conformation with sub-Angstrom RMSD [47]. CCD has been used together with fragment insertion to design a *de novo* loop that alters the substrate specificity of an enzyme [48]. In this work, short backbone fragments were inserted before and after a fixed anchor residue predicted to alter substrate binding followed by CCD to close the chain breaks. This approach produced a design with a four-residue sequence change which was confirmed to be in the correct conformation by X-ray crystallography.

While larger scale backbone motions can be modelled using fragment insertion, more subtle backbone movements are also very important in protein modelling. Natural proteins can be quite tolerant to mutations as the backbone can adjust to accommodate side-chains that would not be permitted using the fixed-backbone approximation. Several methods have been developed to model small backbone perturbations. In many protein design applications, cycles of sequence design followed by potential energy minimisation of the whole structure (including the backbone) are carried out to permit some degree of backbone flexibility. Other methods include extensions to the dead-end elimination algorithm to include backbone flexibility [49]. While these methods result in designs with lower potential energies, they do not always recapitulate the natural sequence variation observed in these proteins. A novel local backbone move called ‘backrub’ was developed after inspecting very high-resolution crystal structures for alternative backbone conformations [50]. This move rotates the backbone around the axis connecting C**α**_i−1_ and C**α**_i+1_, followed by compensating rotations of the C**α**_i−1_ to C**α**_i_ and the C**α**_i_ to C**α**_i+1_ peptide bonds. This results in a shift in the direction of the central side-chain but with minimal changes to backbone hydrogen-bonding geometry. This method was generalised and implemented in the Rosetta software package and, when coupled to sequence design, was shown to significantly improve the recapitulation of experimentally observed sequence variation in protein–protein [51] and protein–ligand [52] interfaces compared with fixed-backbone design methods.

**Figure 2.**
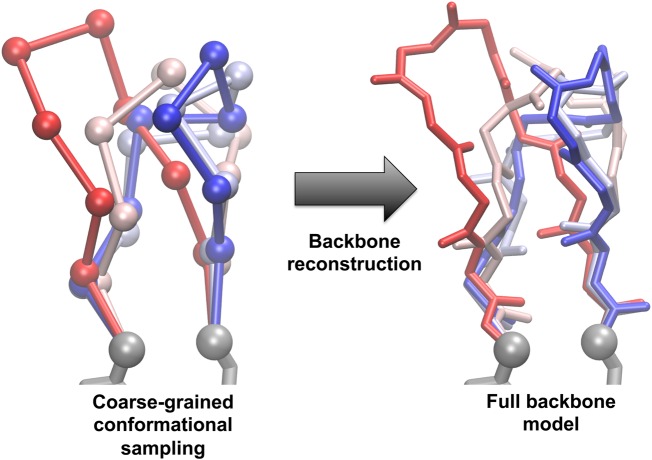
Sampling backbone loop conformations using a coarse-grained model. Conformational space can be rapidly sampled using a reduced representation before being rebuilt into a full-atom model as part of a hierarchical design strategy. The grey atoms are the fixed anchor atoms at the N- and C-terminal ends of the loop being sampled, and the red/blue chains are alternative loop structures.

In the past few years, we have developed new algorithms and software for the fragment-free sampling of backbone loop conformations using a coarse-grained model [53]. This method uses a coarse-grained potential energy function [54] to rapidly sample plausible backbone conformations at the carbon-α level, then accurately reconstructs the full backbone model using a structural alphabet derived using Gaussian mixture models [55]. The potential energy function consists of a pseudo hydrogen-bonding term, a soft steric repulsive term, a pseudo C**α**-C**α** bond term, and local structural terms. The local terms were derived using a structural alphabet and include a pseudo C**α**-C**α**-C**α** bond angle term, a pseudo C**α**-C**α**-C**α**-C**α** dihedral angle term, and reference terms to ensure that the equilibrium distributions of each structural alphabet letter reproduce those observed in a high-resolution training set. An ensemble of loop conformations can be sampled by running successive simulated annealing Monte Carlo trajectories using only local moves that do not propagate down the rest of the chain (Figure 2). When side-chains were added, and the structure was energy gradient minimised using the Rosetta software package, this approach produced results that were equivalent to fragment insertion methods [53]. We propose that the ability to sample directly using coarse-grained potential energy functions enables the efficient incorporation of functional geometric restraints and the use of more sophisticated sampling methods that are more difficult to achieve with fragment insertion methods. Recently, we have successfully applied this fragment-free method to the design of *de novo* backbone protein design [56].

## Conclusion

In the present paper, we have described the methodological advances in computational protein design in the past few decades. Initial approaches to computational design considered only fixed-backbone structures. It has become clear that the incorporation of backbone flexibility is essential to fully explore sequence space and to enable more complex designs. This backbone flexibility can range from small-scale motions, that permit slightly different side-chain orientations, to the large-scale redesign of complete sections of the protein backbone. The rules governing whether a given arbitrary backbone conformation is designable are not well understood. In particular, the computational design of *de novo* backbone loops has proved to be particularly challenging. There has been more success in the design of *de novo* folds composed of secondary structural elements and minimal loops as there are well-understood rules governing the packing of these elements. To date, the vast majority of successful computationally designed functional proteins have relied on fixed-backbone design methods. A better understanding of backbone designability and new design algorithms will enable the complete remodelling of large sections of the protein scaffold resulting in improved enzymes.

## Abbreviation

CCD, cyclic co-ordinate descent.

## Author Contribution

Both the authors contributed to writing this manuscript.

## Funding

This work was funded by the Engineering and Physical Sciences Research Council, UK [grant number EP/K034359/1]. No new data was created during the course of this research.

## Competing Interests

The Authors declare that there are no competing interests associated with the manuscript.
